# Birds may represent a useful animal model for studying human mental disorders

**DOI:** 10.1017/neu.2025.10045

**Published:** 2025-11-05

**Authors:** Anton J.M. Loonen

**Affiliations:** PharmacoTherapy, -Epidemiology & -Economics, Groningen Research Institute of Pharmacy, University of Groningen, Groningen, The Netherlands

**Keywords:** Mechanism mental disorders, avian migration, navigation, habenula, birds, lamprey

## Abstract

In basic neuropsychopharmacological research, some biobehavioural phenomena — *e.g.*, population migration and navigation over long distances — are rarely considered because the most commonly used laboratory animals show little or no evidence of these phenomena. Nevertheless, they can be also relevant for the mechanism of human psychic aberrations. An annual migration is seen in migratory birds, certain marine mammals and several ungulates. For migratory birds, the time of departure is determined by the length of the photoperiod and is much less changeable than the chosen route. When navigating, migratory birds also use the direction and strength of the field lines of the Earth’s magnetic field. Because humans also seem to exhibit a certain sensitivity to the Earth’s magnetic field, the regulation in birds could also provide hints for research on human well-being. Some bird species have such highly developed cognitive abilities that this is considered proof of the possession of consciousness. Therefore, some birds may be suitable as experimental animals in neurobiological models for cognitive functions and for making the world of thought accessible. The dorsal diencephalic conduction system (DDCS) in humans is difficult to study due to its small size and complex architecture, but it is relatively well developed in more primitive vertebrates. For research into the primary interactions between the DDCS and the rest of the brain, the lamprey can be used as laboratory animal. There is manifold evidence that the DDCS along with forebrain and upper brainstem is of functional relevance and the significance of the DDCS in cortical-controlled networks could then be investigated in birds and verified in humans.

## Graphical abstract

**Figure:**
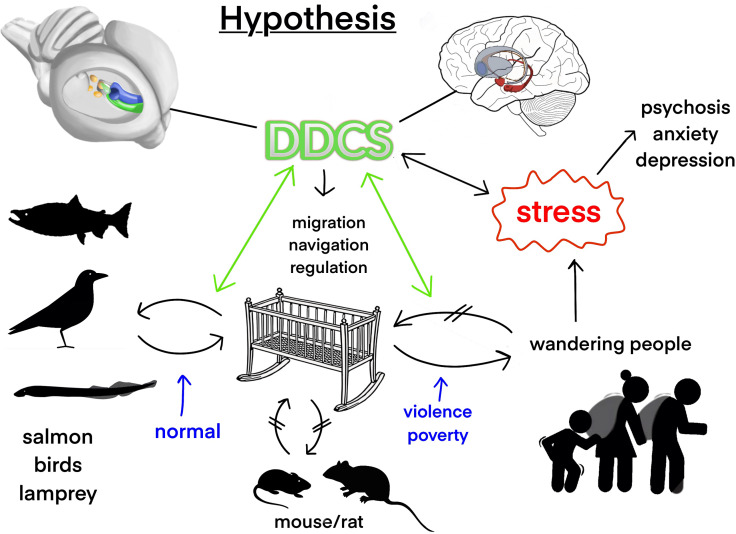

## Summations

- Individuals who are forced to leave their homeland often develop stress-related mental health issues.
- Some birds, turtles, fish, and lampreys return to their birthplace to reproduce.
- The DDCS of birds may play a role in migration/navigation, and also in human mental health issues.

## Considerations

- The anatomy, cytoarchitecture, and connectivity of the dorsal diencephalic conduction system in birds has hardly been studied.
- There may exist significant differences between different bird species.
- The cytoarchitecture of specific forebrain structures in birds and mammals has yet to be compared one-to-one.

## Introduction

During the last half century — that is, since the author’s career as a scientist began — tremendous progress has been made in the development of techniques by which the functioning of the brain can be investigated. This applies to living humans in terms of the development of various neuroimaging and neurostimulation techniques. Groundbreaking work has also been done in the field of (bio)chemical and molecular biological analysis. The foregoing is even more true for technical advances in the field of animal experimental research. Consider, for example, the development of optogenetic research techniques that can very specifically activate or inhibit well-defined components of neuronal circuits (Deisseroth *et al*., 2006; Henderson *et al*., 2009). However, there is one aspect of animal research that has not changed as much: although they have been surpassed by the transgenic mouse (*Mus musculus*) worldwide, purebred strains of the rat (*Rattus norvegicus domestica*, *e.g.*, Wistar rat) is still considered an experimental animal of choice for research in the laboratory. This is hardly surprising; among laboratory animals, the easily bred laboratory rat is incredibly well documented. For most neuroscience experiments, this subspecies has some comparison material available. Such popularity also has limitations. Some biological phenomena cannot be studied in these animals because they do not exhibit them or because they cannot be simulated in an experimental setting. That’s why it’s probably a good idea to use other types of vertebrates as test animals in neuropsychopharmacological experiments.

Recently, a review article was written by the author on the neurobiology of animal migration (Loonen, 2024). For research on this, the rat is actually unsuitable as an experimental animal. Flying and swimming animals have a strong advantage when it comes to migrating, because it costs them much less energy. Population migration is also seen in larger land animals such as some hoofed herbivores (wildebeest, zebra, moose, reindeer), but actually not in small rodents such as the mouse or rat. Mammals basically have three options for surviving scarcity during winter: 1. Seasonal migration, 2. lowering metabolism by heterothermy (torpor and hibernation), 3. or adjusting behaviour to tolerate food scarcity with or without winter stockpiling. Rats belong to the latter category; they do not migrate and are not cardiophysiologically equipped to hibernate (Filatova *et al*., 2023). Although rats seek warmer places to stay (*e.g.*, shelters and houses) in order to cope with the winter cold, true migration over longer distances is not observed. Birds and marine mammals can navigate using the Earth’s magnetic field. An independent sense of direction is seen in rats (Poucet *et al*., 2015), but to the author’s knowledge it has not been demonstrated that the Earth’s magnetic field plays a role here (Shirdhankar & Malkemper, 2024).

Nevertheless, it should not be forgotten that modern man originally led a nomadic existence as hunter-gatherers. The domestication of animals began about 15,000 years ago (Zeder, 2012), and the first permanent agricultural settlements of humans occurred during the following ‘Neolithic revolution’ some 10,000 years ago (Shavit & Sharon, 2023). So that’s actually very recent in human’s evolution. Therefore, studying the neurobiological mechanisms that regulate animal migration may also be relevant for understanding the (patho)physiology of, *e.g.*, human wandering. Perhaps remnants of this can be found in the physiology of present-day humans and play a role in seasonal fluctuations in the severity of mood disorders (Dollish *et al*., 2024). Moreover, these mechanisms may also contribute to the amplified occurrence of individuals with schizophrenia (Selten *et al*., 2020) and other mental disorders (Osman *et al*., 2024) among those who migrate in modern times.

With this article, the author aims to draw attention to the possibility of using animals from classes other than *Mammalia* (in particular *Aves*) as experimental subjects in neuropsychopharmacological studies. He aims to substantiate this by outlining how various aspects of the neurobiological mechanisms underlying migration and navigation in animals may also be relevant to human behaviour. As a prerequisite for application in this context, the anatomy of the forebrain of birds must be compared in more detail with that of humans. For research into the function of the dorsal diencephalic conduction system (DDCS), birds are less suitable similar as humans. It is better to turn to the most primitive classes of vertebrates, because in these the DDCS is relatively large and less complex. Of note, previously the term “connection” rather than “conduction” was used because it better reflects the complex role of the habenula as a reciprocal connecting hub, but “conduction” is the original term (Sutherland, 1982). To avoid unnecessary repetition, the author refers to previous articles (Loonen & Ivanova, 2018a, 2018b; Loonen, 2024) for a more detailed description and the correct references.

## Animal migration

### Timing of migration in birds

Of all animal species that undertake annual migrations, migratory birds are by far the best studied (Loonen, 2024). Millions of birds change residence each spring and fall between their wintering grounds and their higher latitude breeding grounds. The physiological changes that occur in migrants compared to conspecifics that breed in the wintering grounds (residents) have all been very well elucidated. The main determinant of timing is the ratio of day to night hours, *i.e.,* the length of the photoperiod (Stevenson and Kumar, 2017). The circannual rhythm is regulated in migratory birds by a photosensitive centre in specific regions of the hypothalamus (Kumar *et al*., 2004; Natesan *et al*., 2002). These photosensitive neurons are coupled neither to the retina nor to the epiphysis and project onto the median eminence and the part of the adenohypophysis in close apposition to it: the pars tuberalis (PT) (Korf, 2018). These photosensitive neurons activate a system that ultimately regulates the secretion of Gonadotropin-Releasing Hormone (GnRH) by specific hypothalamic neurons into the primary portal hypophysial circulation in the median eminence. The process involves PT-specific cells in the pars tuberalis and particular glial cells (tanycytes) lining the third ventricle at the median eminence and mediobasal hypothalamus. An essential role is played by thyroid stimulating hormone (TSH-β) secreted by PT-specific cells that induces these tanycytes to express type 2 and type 3 deiodinase (Dio2, Dio3) within the basomedial hypothalamus (Korf, 2018; Dardente & Migaud, 2021). In spring, Dio2 predominates, producing the active lyothyronine (T3) and at the end of summer Dio3 producing the inactive reverse trijiodothyronine (rT3) and diiodothyronine (T2). In parallel, GnRH release is promoted in spring and extinguishes at the end of summer. This sexual maturity (particularly in females) determines migration to and from the breeding grounds (Kimmitt, 2020).

Mammals also display this system in the ventral part of the wall of the third ventricle, but the photosensitive neurons in the hypothalamus are missing (Korf, 2018; Dardente & Migaud, 2021). Instead, the secretion of TSH-β is regulated by type 1 melatonin receptors (MT1) and depends on the secretion of the hormone melatonin by the pineal gland. The pineal gland is connected to photosensitive cells of the retina and produces melatonin at an inverse photoperiod-dependent rate. The relationship with reproduction is preserved in some mammals (sheep, goat, hamster), but the system is also preserved in species where this is less (F344 rats) or not (mouse) the case (Dardente & Migaud, 2021).

When considering this endocrine regulation of migration timing, a comparison with the add-on treatment of human mood disorders comes to mind (with light, melatonin agonists, lyothyronine, and oestrogens) (Loonen & Ivanova, 2016a). It would also be interesting to conduct experimental studies in birds to investigate whether the response to pharmacological interventions depends on this circadian regulation of migratory behaviour and then translate this to treatment of humans.

### Migration in mammals

Some mammals also exhibit seasonal migration. The catchiest examples of this are certain whales, which travel relatively long distances between low latitude breeding and high latitude foraging areas where they spend winter and summer, respectively (Andrews-Goff *et al*., 2018; De Weerdt *et al*., 2023). Because relatively little food is available in the intermediate area, whales, like some songbirds, make stopovers precisely to brush up on their feeding status (Silva *et al*., 2013).

Other animals that migrate depending on the seasons are the large hoofed mammals, ungulates, like caribou also known as (aka) reindeer (*Rangifer tarandus*), moose (*Alces alces*), wildebeest aka gnu (*Connochaetes taurinus*), and zebra (*Equus quagga*). In these animals, the reasons for this seasonal migration are: 1. the availability of higher value food, 2. the one-sided availability of essential nutrients and 3. the escape from seasonal predators, parasites and insects (Bolger *et al*., 2008). Evading predators is, of course, relative: migrating herds are often also followed by predators such as grey wolf (*Canis lupus*) that also benefit from improved nutritional status that way (Joly *et al*., 2019). Actually, humans can also be included in this category of active followers. This is most true of the Indigenous people who inhabited the great plains of North America and hunted the wandering herds of bison (*Bison bison*). More domesticated were reindeer in the case of the original inhabitants of Lapland, the Sami (or Samen). But even in this case, humans followed the herds as they migrated and not the other way around. An interesting phenomenon that occurs in these ungulates is that they apparently anticipate a later need for nutritious food. This is nicely illustrated by some caribou in Newfoundland that begin their spring migration even before the snow has largely melted away to be in an area with plenty of nutritious young greenery when they later calve and need to lactate (Laforge *et al*., 2021). Apparently in these animals, the melting of the snow is a cue to initiate migration. Although less explicitly than in migratory birds, reproduction also plays an important role in these ungulates as a reason for migration. The wildebeest are also known to tailor their annual migration to rainfall and places where food is most nutritious (Boone *et al*., 2006; Holdo *et al*., 2009). The latter is called following the green wave (Aikens *et al*., 2017). However, on which internal and external cues they initiate this journey is not well known. Most likely, this is based on a cognitive process that can be incorporated into general behavioural planning.

### The importance of social stimuli

The timing of migration is dependent not only on internal cues — such as the secretion TSH-β leading to that of testosterone (Sharma *et al*., 2022) or the secretion of Ghrelin (Lupi *et al*., 2022) — and external cues (such as the melting of snow (Laforge *et al*., 2021)), but to a significant extent also on social cues (Guttal & Couzin, 2010; Oestreich *et al*., 2022; Reyes & Szewczak, 2022). Oestreich and colleagues (2022) distinguish six mutually non-exclusive types of social interactions between conspecifics that determine this timing in different animal species (mainly vertebrates). Following this, it can be postulated that the timing of migration is controlled by three concurrent processes embedded in a reasonably flexible cognitive procedure: 1. the perception and response to internal and external cues by (any) trail seeking individuals; 2. the social interactions between these trail seeking individuals and the rest of the population, and 3. the added value that the social interactions bring to the adequate and specific response to internal and external cues of the trail seekers. This timing and possibly the route and destination can be adapted to climate change and human-induced barriers by influencing some of the individuals as previously suggested in Loonen (2024). Such changes in migration behaviour may also occur spontaneously. This may be concluded from research on short-term changes in the breeding area and route taken by the pink-footed goose (*Anser brachyrhynchus*) (Madsen *et al*., 2023).

## Animal navigation

### Route determination

The study by Madsen *et al*. (2023) reveals another insight: apparently, the choice of route is variable. The original flight route of the pink-footed goose was from northern Denmark across Norway to Svalbard in the Arctic Ocean, some 565 km north of Norway. As this route became less attractive, some animals flew a stretch with the migration route of the taiga bean goose (*Anser f. fabalis*) over Sweden and its staging areas on the Swedish Bothnian coast to its breeding grounds in northern Fennoscandia. Some pink-footed geese previously deviated from this flight route during spring migration to fly to their original breeding grounds on Svalbard, but others flew on to a new breeding area in Novaya Zemlya in northern Russia. The latter group increased in 10 years to 3000 (spring)-4000 (autumn) specimens (Madsen *et al*., 2023). That social interactions with conspecifics are very important for juvenile migration is also shown by the research of Loonstra *et al*. (2023). They machine-hatched eggs from nests of the black-tailed godwit (*Limosa limosa limosa*) and hand-raised these specimens. In autumn they released siblings in the area of origin (Netherlands) or 1000 km east (Poland) and monitored the route they chose. It turned out that for the most part they followed the route of the local birds in the area where they were released. Interestingly, something similar may also be the mechanism by which the pink-footed goose got to Novaya Zemlya (Madsen *et al*., 2023). Some taiga bean geese undertake a molt migration from Finland to Novaya Zemlya and pink-footed geese may have flown with them. In this way, the new breeding area may have been actively discovered by these birds.

The multi-annual variability of bird migratory movements shows that the route chosen is much less precisely fixed than the timing of departure (Stanley *et al*., 2012). Inter-individual variability is also significant (Pancerasa *et al*., 2022). This constitutes an indication that the emotional and cognitive interpretation of variable sensory information plays a greater role in route determination than in departure timing. It appears that the route is mainly calculated by means of a cognitive neural process and that the timing of departure is mainly regulated by a neuroendocrine emotional process (Loonen, 2024). Migratory birds combine information about the position and strength of magnetic field lines with that about the position of celestial bodies and that of the landscape (Åkesson and Bianco, 2017; Muheim *et al*., 2018).

### Sensitivity to earth’s magnetic field

A large number of living creatures are sensitive to the Earth’s magnetic field, *i.e.,* exhibit magnetoreception; these include plants (Galland & Pazur, 2005), microorganisms (Lin *et al*., 2020; Monteil & Lefevre, 2020), insects (Fleischmann *et al.*, 2020), fish (Formicki *et al*., 2019; Naisbett-Jones & Lohmann, 2022), birds (Mouritsen & Ritz, 2005; Wiltschko & Wiltschko, 2019) and several species of mammals (Walker *et al*., 1992; Kremers *et al*., 2014; Caspar *et al*., 2020; Zhang & Malkemper, 2023). Several indications exist that humans too are capable of magnetoreception (Baker, 1987; Carrubba *et al*., 2008; Foley *et al*., 2011; Wang *et al*., 2019; Chae *et al*., 2022), but that interference with this perception occurs by low-level anthropogenic electromagnetic fields and leads to medical problems is controversial (Henshaw & Philips, 2024). Two forms of magnetosensing are involved in birds: one via cryptochrome and the other via magnetite (Johnsen & Lohmann, 2005; Mouritsen & Ritz, 2005; Clites & Pierce, 2017; Wiltschko & Wiltschko, 2019). However, these theories are recently heavily criticised by Panagopoulos *et al.* (2024), who argue that specific magnetosensitive organs are not needed at all to enable sensitivity to the geomagnetic influence. This will be left aside here.

Cryptochromes belong to a family of photolyases/cryptochromes (PHR/CRY) that are very widespread in living nature and are involved in a wide range of processes (Ozturk, 2017). They are flavin adenine dinucleotide (FAD)-bearing flavoproteins (Calloni & Vabulas, 2023), which in vertebrates also play a role in repression of genetic expression and in reentrainment of the circadian clock (Parico & Partch, 2020; DeOliveira & Crane, 2024). Of particular interest are cryptochrome 1 (Cry1a and Cry1b), cryptochrome 2 (cry2) and cryptochrome 4 (Cry4a and Cry4b) and these are found in birds mainly in the retina and also in the pineal gland (Nagy & Csernus, 2007; Rotov *et al*., 2022; Wiltschko & Wiltschko, 2019). The sensitivity to the Earth magnetic field is attributed to the difference in the spin of unpaired electrons upon the recoil of FADH· radicals from the FAD cofactor of these cryptochromes (Hore and Mouritsen, 2016; Karki *et al*., 2021; Zhang & Malkemper, 2023; DeOliveira & Crane, 2024). The presence in cones in the retina would thus make it sensitive to the direction of the field lines of the geomagnetic field, which in turn depends on its position on Earth (Mouritsen & Ritz, 2005; Rotov *et al*., 2022; Wiltschko & Wiltschko, 2019; Wiltschko *et al*., 2021).

Magnetite involves very small particles (called nanoparticles) of iron (II, III) oxide (Fe_3_O_4_), which can orient themselves in a magnetic field (Cadiou & McNaughton, 2010; Shaw *et al*., 2015). Magnetite crystals of 50-100 nm are permanently magnetised and are called “magnetic single-domain” particles. Assemblies of crystals up to 50 nm are “superparamagnetic” particles, that is, they are magnetisable by an external magnetic field but lose this property when this field is absent (Cadiou & McNaughton, 2010). These particles are found in nerve endings in the skin over the rim of the upper beak of homing pigeons (Fleissner *et al*., 2007), belonging to the ophthalmic nerve branch of the trigeminal nerve (Heyers *et al*., 2010). The specialised epithelium of the lagena of the inner ear has also been mentioned as a possible carrier of magnetoreceptors (Wu & Dickman, 2011), but later research found no evidence for the presence of magnetite in this structure (Malkemper *et al*., 2019). Magnetite-containing receptors could be sensitive to geomagnetic field strength and incorporate this information into a map of the landscape.

Little research has been done into whether and what role the perception of the Earth’s magnetic field plays in regulating neurochemical and neuropharmacological processes in humans. To distinguish this sensitivity from that for other types of magnetic fields, we could perhaps look at how this works in birds.

## Comparison of the avian to the mammalian cerebrum

Birds can be quite good models for certain processes that also occur in humans. However, a major problem exists: the forebrain is built differently and the differences were interpreted differently in the first half of last century (Loonen, 2024). Reiner *et al.* (2004) describe that it was not until 2002 that a new nomenclature was adopted in which the misleading concept that the avian hemispheres consist almost entirely of striatal areas was abandoned.

### Joint ancestry

Birds and mammals are both descended from the same extinct *captorhinomorphs* (Butler, 1994). From this ancestor developed synapsids on the one hand and non-synapsids (diapsids) and turtles on the other. All reptiles (except turtles) evolved from the diapsid group and birds are a late offshoot of this group. From the synapsids later emerged the mammals. In birds and mammals, the forebrain developed differently (Butler, 1994). In mammals a lining emerged with a six-layered neocortex and in birds this became a dorsal ventricular ridge (DVR) and the so-called Wulst with a different structure. Although they grew differently in birds and mammals, all structures of the *captorhinomorph* ancestor are found in the forebrain of both birds and mammals.

### Anatomical differences

**Figure 1.**
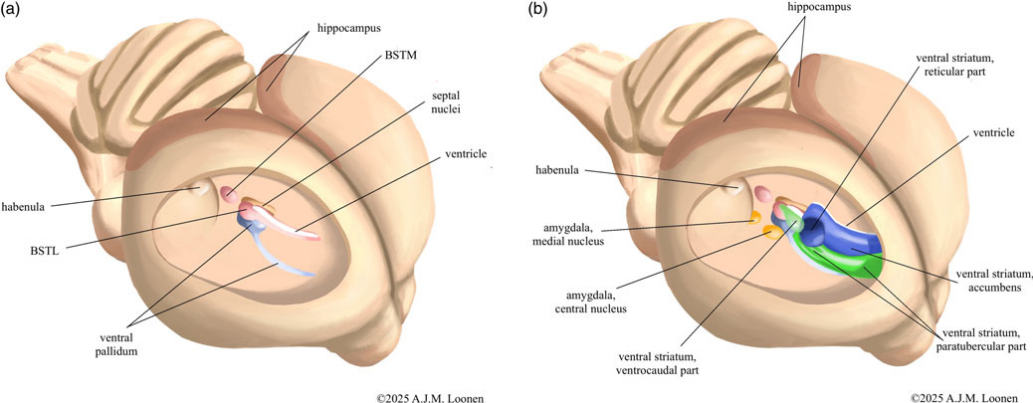
Insight view into the right hemisphere of pigeon’s brain with schematic representation of the amygdaloid and ventral pallidum (A) and striatopallidum (B). The bed nucleus of the stria terminalis is shown here as a separate amygdaloid pallidum and the nuclear amygdala as a separate amygdaloid striatum, but in reality these structures are cytologically and anatomically highly intertwined. Parts of the medial striatum that may also be homologues of the core and shell portion of the ventral striatum are not shown. The paratubercular and ventrocaudal striatal regions can be considered the shell of the ventral striatum. The nature of the shown reticular portion of the ventral striatum is uncertain. BSTM - medial bed nucleus of the stria terminalis, BSTL - lateral bed nucleus of the stria terminalis. Adapted from figures by Bruce *et al*. (2016) and Medina *et al*., 2023.

The greatest differences in blueprint exist at the forebrain level and involve the basal ganglia, dorsal thalamus, and the so-called pallial structures; as described in detail by the author in an earlier article (Loonen, 2024). Paraphrased in simpler terms, the differences boil down to the fact that in birds the forebrain structures involved in the processing of visual, acoustic and balance sensory information (collothalamic) are developed more than those for somatosensory and visceral data (lemnothalamic) (Butler, 1994). In mammals, this development does not appear to be that unilateral. It is easy to imagine how this translated into functional differences. Regarding the basal ganglia, gross anatomical differences exist mainly in the dorsal striatopallidum (Kuenzel *et al*., 2011). Anatomically, the amygdaloid and ventral striatopallida of birds and mammals are apparently reasonably similar (Abellán & Medina, 2009; Bruce *et al*., 2016). To illustrate this, the relative position of these phylogenetically older structures in the ventral hemisphere is shown schematically in Fig. 1. More differences are described at the microscopic level (Abellán & Medina, 2009; Kuenzel *et al*., 2011; Bruce *et al*., 2016; Medina *et al*., 2023), but the author found no direct, one-to-one comparative studies examining the extent of these differences. As for tissues derived from the pallium, the differences are quite significant (Medina *et al*., 2023). This includes structures that do not derive from the dorsal pallium, such as the corticoid amygdala (Hanics *et al*., 2017; Medina *et al*., 2023) and the hippocampus (Atoji *et al*., 2016; Striedter, 2016) (see Fig. 1). So this goes far beyond just for DVR and Wulst (Belgard *et al*., 2013).

### Functional similarities

Despite these anatomical differences between mammals and birds, relevant similarities exist at a more fundamental level in the wiring that plays a role in the function of different brain structures (Atoji *et al*., 2016; Güntürkün *et al*., 2021; Jarvis *et al*., 2013; Smulders, 2017; Herold *et al*., 2019). It are precisely these similarities despite all anatomical differences that make birds particularly interesting as experimental animals. Some bird species are remarkably intelligent (Bugnyar, 2024; Güntürkün *et al*., 2024). As indicated in section 3.1, navigational ability is a highly developed cognitive skill in various bird species. Contrary to previous thinking (see Nevitt and Hagelin, 2009), the sense of smell is also significantly developed in at least some bird species (Prada & Furton, 2018; Gagliardo & Bingman, 2024). This is not very different from humans, where smell as a sense also co-determines emotional behaviour (Rolls, 2019; Bratman *et al*., 2024) and also plays a role in cognitive functioning (Green *et al*., 2023; Cai *et al*., 2024). Therefore, it might be an interesting challenge to use birds as experimental animals in biobehavioural research questions, including those related to cognitive functioning and consciousness.

## Dorsal diencephalic conduction system (DDCS)

### Relevance of using birds in studying DDCS

At first glance, birds seem less suitable as experimental animals for investigating the role of the dorsal diencephalic conduction system (DDCS) in regulating anxiety and mood (Loonen & Ivanova, 2018a, 2018b, 2019a; Loonen *et al*., 2021) and in addiction (Loonen *et al*., 2016; Batalla *et al*., 2017; Loonen, 2024). This system connects various structures of the forebrain to important monoaminergic and cholinergic centres of the upper brainstem (Batalla *et al*., 2017; Metzger *et al*., 2021; Loonen & Ivanova, 2022). Via ascending efferent fibres, these upper brainstem centres in turn selectively regulate the activity of the various components of the forebrain. Along these lines, the DDCS determines response flexibility in social, defensive, and appetitive contexts. A well-known example is the role of fibres running from the pallidum to the lateral habenula that regulate whether behaviour continues or is aborted in lampreys (Stephenson-Jones *et al*., 2013). These fibres — which are known to co-transmit glutamate and GABA (Meye *et al*., 2016; Kim & Sabatini, 2023; Shabel *et al*., 2014) — probably play a similar role in obsessive–compulsive disorder (Loonen & Ivanova, 2019b). A problem in studying the role of the DDCS in regulating human behaviour is that in humans this system is very small and still complex in composition. The habenula consists of a lateral (∼ 94%) and a medial (∼ 6%) division (Díaz *et al*., 2011) and in humans measures only 15–30 mm^3^ on each side of the midline (Batalla *et al*., 2017; Loonen & Ivanova, 2022). Nevertheless, the habenula consists of a large number of different nuclei and regions that differ in terms of connections and chemoarchitecture (Loonen & Ivanova, 2022). To the best of my knowledge, the DDCS of birds is still chronically understudied in comparison to other classes of animals. There is some evidence for the presence of major components of the DDCS in birds (*e.g.*, Medina & Reiner, 1994, 1997). However many important details about the anatomy and chemoarchitecture of this system in birds still need to be clarified. Other important details of the DDCS such as the exact course of the fasciculus retroflexus, which is the bundle of fibres connecting the habenula to the brainstem have only been described recently (Ferran & Puelles, 2024). Thus, it is important to further investigate the avian DDCS as well as to determine its role in navigation and magnetosensing (Loonen, 2024), because these functions may be less developed in mammals used as animal models for mental diseases. Any residues of this avian functionality in humans can then be better targeted and tested out.

### Possible alternative as experimental animal

The DDCS is phylogenetically very old. The habenula is already present in an evolutionary ancestor of the first vertebrates (Loonen, 2024). These first vertebrates from 560 million years ago had a central nervous system (CNS) similar to that of the present-day lamprey. Although relatively small compared to the rest of the CNS, the forebrain of the lamprey is composed of the same components as the forebrain of humans, although of course the proportions have considerably changed and several ‘newer parts’ have developed (Loonen & Ivanova, 2015, 2016b). The DDCS occupies a relatively large portion of the forebrain in the lamprey (Robertson *et al*., 2014; Grillner, 2021). This makes it interesting to investigate the mono- and polysynaptic afferent and efferent part of the DDCS under the use of advanced neuropharmacological research techniques. The anatomy and cytoarchitecture can indeed be studied well in the relatively simple brain of the lamprey. However, the precise function is more difficult to determine because DDCS’s possible significance in behavioural experiments has not yet been fully explored. The precise significance of this connectivity can, therefore, better be verified in zebrafish (*Danio rerio*). It should be noted, however, that these ray-finned fish have an everted instead of inverted forebrain which changes the anatomical relationships (Mueller, 2022; Nieuwenhuys, 2009). Zebrafish are an excellent model for studying the genetic, neurochemical and pharmacological underpinnings of mental disorders (Meng *et al.*, 2025) such as anxiety disorders (Golushko *et al*., 2025) and autism (Jiao *et al*., 2024). Furthermore, the involvement of the habenula in zebrafish in such processes has been investigated previously (Agetsuma *et al*., 2010; Andalman *et al*., 2019). By combining this with findings in lampreys, the mechanism can be refined further. This author has previously suggested, quite rightly, that the lamprey should also be recognised as a suitable experimental animal for the regulation of human behaviour (Loonen & Ivanova, 2018a).

## Why recommend using birds and lampreys in animal models for human mental disorders?

The question may arise as to why it is at all interesting to look at the brains of “early” vertebrates such as lampreys and non-mammalian “late” vertebrates such as birds in order to gain insight into the pathophysiology and pharmacology of human mental disorders. Its relevance may lie in the presence or absence of consciousness. Now I want to steer clear of the age-old discussion of what consciousness actually is, which takes place in a combination of neuroscientific and philosophical domains (León & Zahavi, 2023; Wagner-Altendorf, 2023; Kozuch, 2024). For this article, I want to limit myself to the personal view that consciousness is the human mind capacity that enables the individual to perceive and define oneself by translating perceptions of the external world into strictly personal thoughts and sensations. According to the classical view, a properly functioning cerebral cortex is essential for having and maintaining consciousness (Koch, 2018; Nieder, 2021). However, some birds — members of the corvid songbird family (crows, ravens, jays) — exhibit cognitive abilities that also indicate the presence of consciousness (Güntürkün, 2021; Nieder, 2021). Reptiles and amphibians do not have these and thus probably do not have consciousness (Nieder, 2022). In humans, consciousness is at least partly responsible for the onset and manifestations of mental disorders. However, the cerebral cortex (neocortex, isocortex) of humans is so extensive that the significance for the emergence of specific mental disorders has a large number of degrees of freedom. This makes the interpretation of large neuroimaging studies aimed at finding cortical abnormalities in, for example, individuals with schizophrenia (Van Erp *et al*., 2018) or depression (Schmaal *et al*., 2017) extremely difficult. The lack of an advanced and well-developed dorsal pallium makes the cognitive interpretation of sensory information impossible. It may be assumed therefore that “early” vertebrates such as the lamprey perceive the external world by having emotional sensations such as fear, pain, gloom without the accompanying awareness. According to this author, this makes it likely that these emotions are also generated in humans in the so-called ‘primary’ forebrain (amygdaloid and hippocampal complexes, the hypothalamus, septal nuclei, and habenula). Indeed, lampreys must be able to perceive the external environment, because they are able to respond appropriately to an unsafe environment. Neurobiology can be examined in these early vertebrates without the modulation (and sensation) by the cerebral cortex that occurs in humans. At the other end of the vertebrate spectrum, if crows have - if only partially – human-advanced cognitive abilities (Bugnyar, 2024; Güntürkün *et al*., 2024) that in humans are related to consciousness (Güntürkün, 2021; Nieder, 2021) it is likely that their far-through evolved dorsal pallium can affect the “primary” brain in the same way as the human isocortex. By comparing the modulation observed in humans with that in birds, it may be possible to distinguish specific cortical influences via forebrain networks from non-specific influences. This is indeed an interesting thought that is worth investigating further.

## Statement

During the preparation of this work the author used DeepL Translate (https://www.deepl.com/nl/translator) in order to increase variability of English language formulations. After using this tool/service, the author reviewed and edited the content as needed and takes full responsibility for the content of the publication.
